# Supplementary material for: The Use of Flavylium Salts as Dynamic Inhibitor Moieties for Human Cb5R
Source: Molecules. 2022 Dec 23;28(1):123. doi: 10.3390/molecules28010123 (PMC9822168; doi:10.3390/molecules28010123)
Supplement: Supplementary file 1 [file molecules-28-00123-s001.zip › molecules-2040681-supplementary.pdf]

Supplementary Information

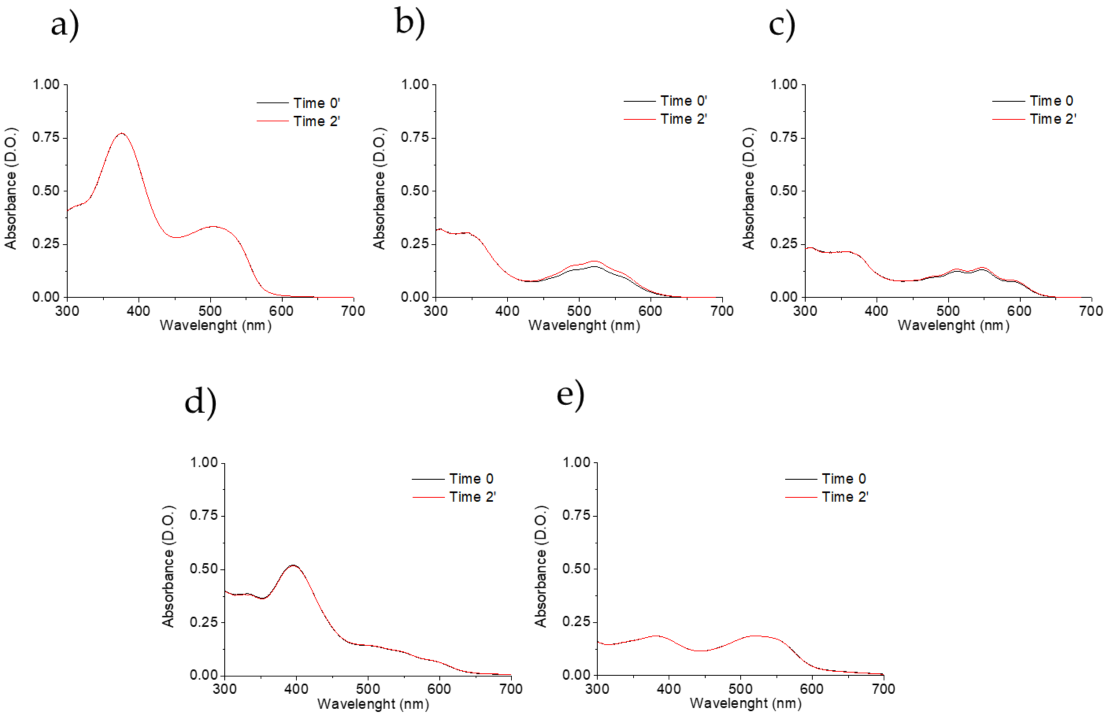

- Figure **S1**. Absorbance spectra of the flavylium salts (50 μM) were immediately measured after addition to potassium phosphate buffer 20mM, EDTA 1mM, pH 7.0, and after incubation for 2 minutes in these conditions. The absorbance spectra of compounds 1, 2, 3, 4, 5 measured in phosphate buffer, EDTA 1mM pH 7.0 are shown in panel a, b, c, d, e, respectively, after addition to the buffer (black line) and after 2 minutes incubation (red line).

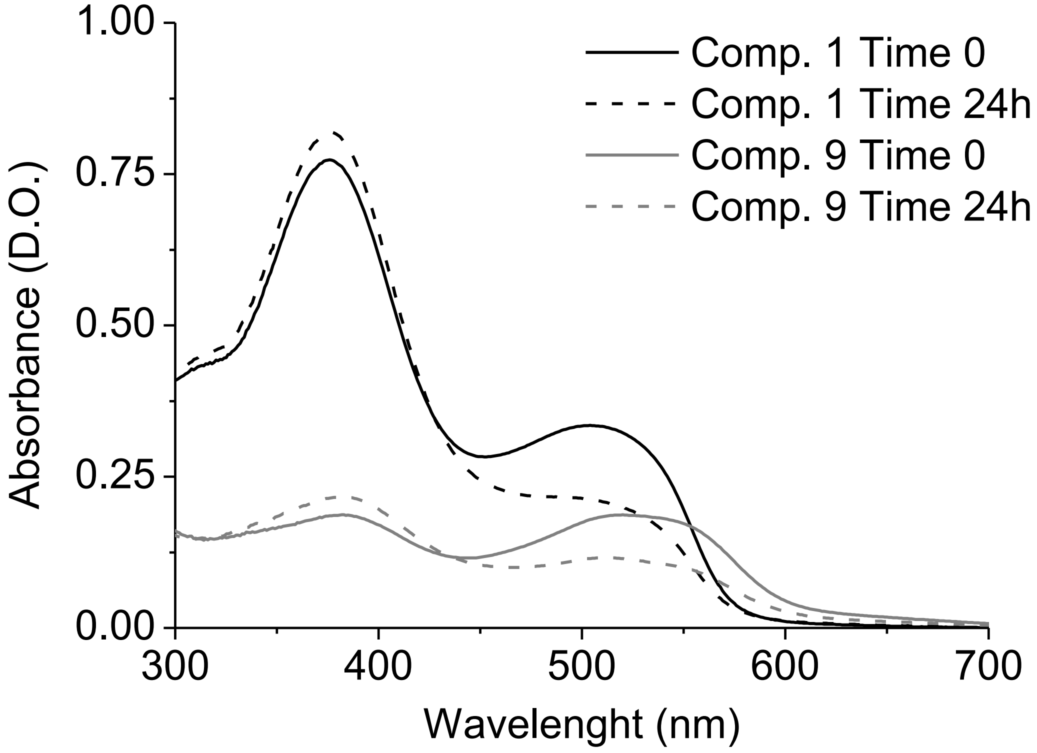

- Figure **S2**.The UV-vis spectra of the non-equilibrated form of compounds 1 and 5 were obtained after instant measurement of the spectra of the acidified solution of each compound (DMSO+ 0.1M HCl) upon addition to 100mM of potassium phosphate pH 7.0 (non-equilibrated conditions) or by incubation in this buffer for 24h (equilibrated conditions), confirmed that the composition of the tested aliquots was correct (Panel c).

Q6BCY4	1	M N S R R R E P I T	L Q D P E A K Y P L	P L I E K E K I S H	N T R R F R F G L P	40
1I7P	27	. . M H H H H M I T	L E N P D I K Y P L	R L I D K E I L S H	D T R R F R F A L P	64
Q6BCY4	41	S P D H V L G L P V	G N Y V Q L L A K I	D N E L V V R A Y T	P V S S D D D R G F	80
1I7P	65	S P Q H I L G L P I	G Q H I Y L S T R I	D G N L V I R P Y T	P V S S D D D K G F	104
Q6BCY4	81	V D L I I K I Y F K	N V H P Q Y P E G G	K M T Q Y L E N M K	I G E T I F F R G P	120
1I7P	105	V D L V V K V Y F K	E T H P K F P A G G	K M S Q Y L E N M N	I G D T I E F R G P	144
Q6BCY4	121	R G R L F Y H G P G	N L G I R P D Q T S	E P K K T L A D H L	G M I A G G T G I T	160
1I7P	145	N G L L V Y Q G K G	K F A I R A D K K S	N P V V R T V K S V	G M I A G G T G I T	184
Q6BCY4	161	P M L Q L I R H I T	K D P S D R T R M S	L I F A N Q T E E D	I L V R K E L E E I	200
1I7P	185	P M L Q V I R A V L	K D P N D H T V C Y	L L F A N Q S E K D	I L L R P E L E E L	224
Q6BCY4	201	A R T H P D Q F N L	W Y T L D R P P I G	W K Y S S G F V T A	D M I K E H L P P P	240
1I7P	225	R N E H S S R F K L	W Y T V D K A P D A	W D Y S Q G F V N E	E M I R D H L P P P	264
Q6BCY4	241	A K S T L I L V C G	P P P L I Q T A A H	P N L E K L G Y T Q	D M I F T Y	276
1I7P	265	G E E T L I L M C G	P P P M I Q F A C L	P N L E R V G H P K	E R C F T F	300

- Figure **S3**. Alignment of the rat Cb5R sequence of the PDB used in this study (PDB file:1I7P and the sequence of human soluble Cb5R (Unitpro code: Q6BCY4). Cb5R's residues that were found common for the interaction with inhibitors are labeled in orange. As shown in the alignment, those residues are the same in the sequence of human Cb5R.

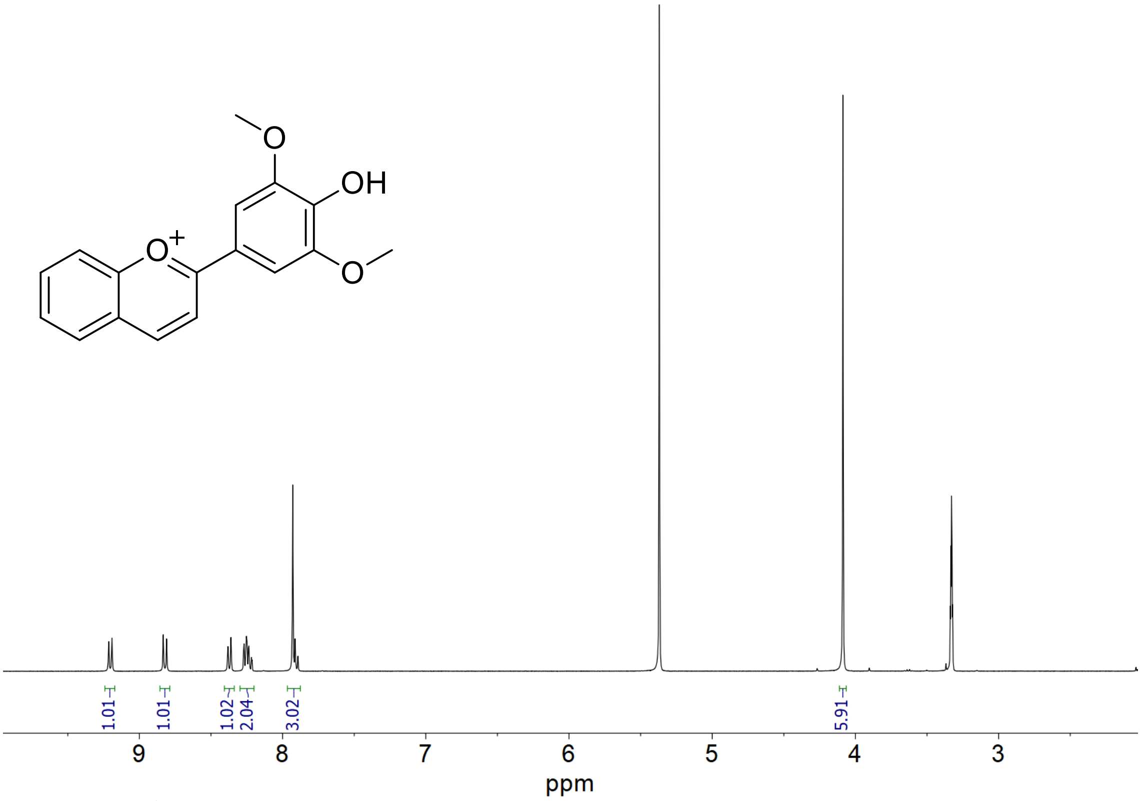

Figure S4. ^{1}H NMR spectrum (400 MHz) of compound **3** in 99:1 (v:v) CD_3OD:DCl 20% in D_2O.

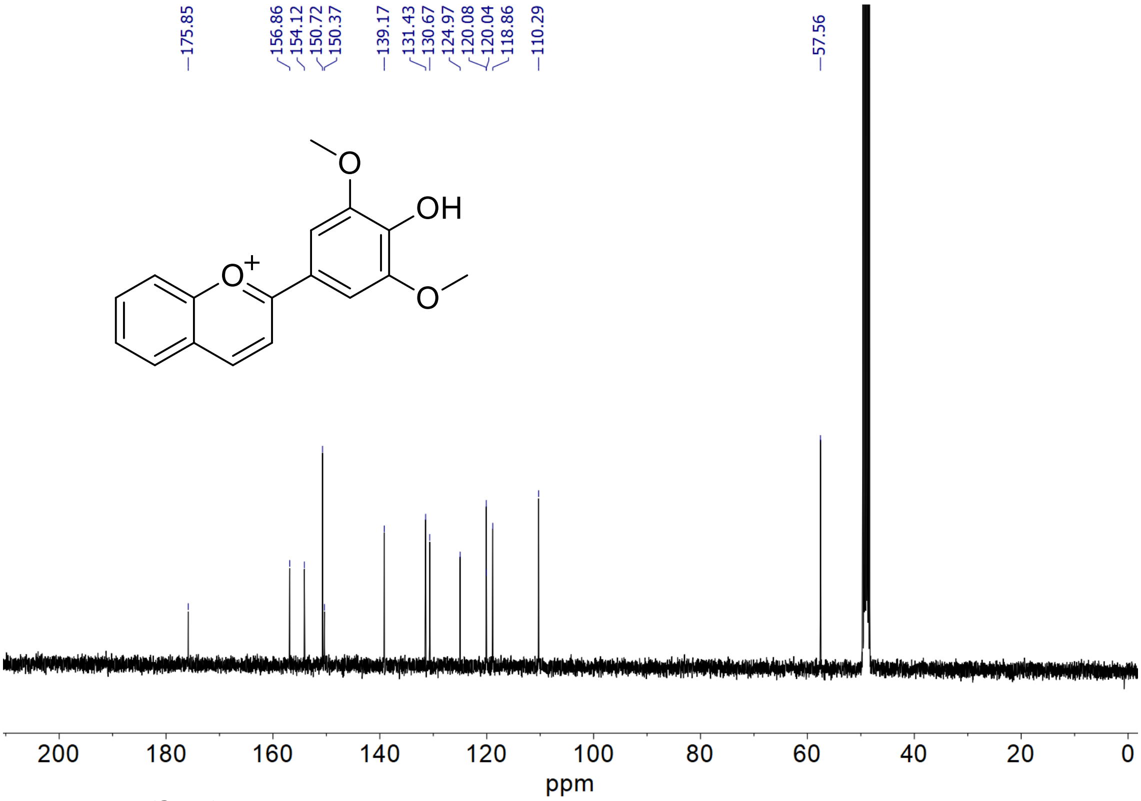

Figure S5. $^{13}C\{^{1}H\}$ NMR spectrum (101 MHz) of compound **3** in 99:1 (v:v) CD_3OD:DCl 20% in D_2O.

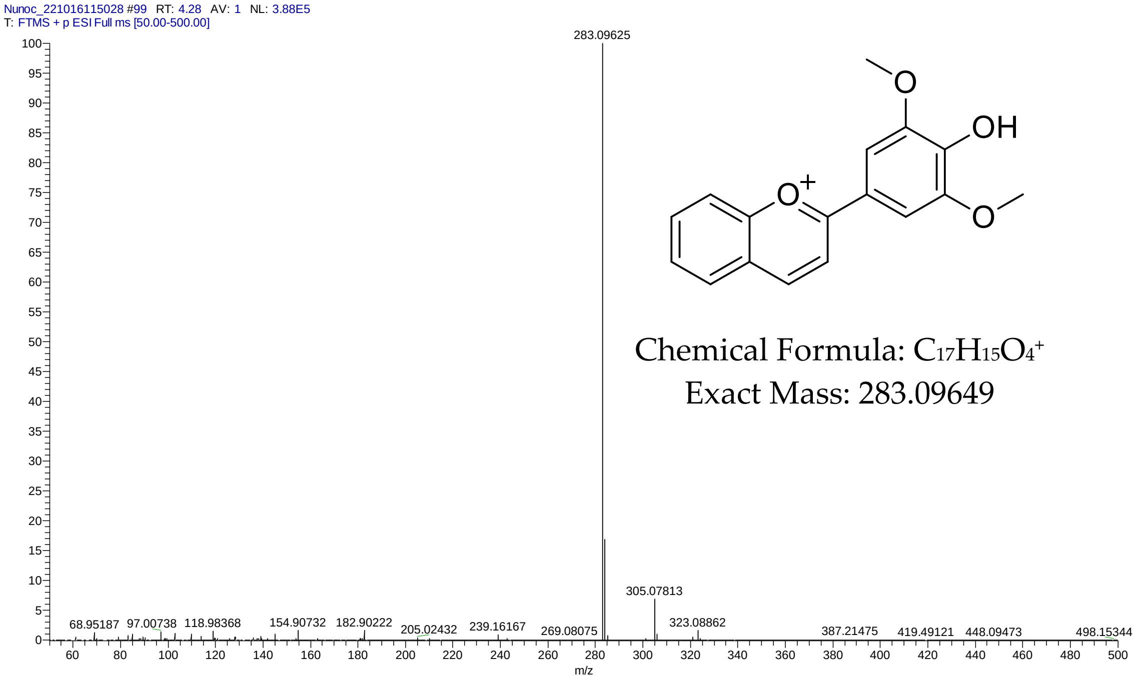

Figure S6. HRMS spectrum of compound **3**.
